# A Case Report of a Novel Alpha-Synuclein Vaccine (TRB-001) in a Parkinson’s Patient: Safe Administration and Induction of a High-Titer, High-Avidity Functional Antibody Response

**DOI:** 10.3390/vaccines14060466

**Published:** 2026-05-23

**Authors:** Dieter Volc, Caroline Thun-Hohenstein, Sabine Schmidhuber, Markus Mandler, Achim Schneeberger

**Affiliations:** 1 Atomos Clinic Waehring, 1180 Vienna, Austria; 2 Tridem Bioscience, 1030 Vienna, Austria

**Keywords:** Parkinson’s disease, vaccination, alpha-synuclein, immunotherapy

## Abstract

Background/Objectives: Parkinson’s disease (PD) is a major neurodegenerative disorder with no cure. The goal is to develop an active immunotherapy targeting aggregated alpha synuclein (aSyn), the root cause of PD. TRB-001 is the lead candidate of a novel class of vaccines. It is a peptide/protein conjugate coupled to sugar residues, which is used to target and activate antigen-presenting cells, and addresses aSyn. Methods: A 33-year-old male, diagnosed with PD seven years previously, with a Hoehn & Yahr stage of 1, taking Levodopa/Benserazide (100/25 mg, 6× per day), Rotigotine (8 mg) and Rasagiline (1 mg), amounting to a Levodopa equivalent daily dose (LEDD) of 940 mg, also complicated by impulse control disorder, requested experimental therapy. He received a total of four TRB-001 administrations (weeks 0, 4, 8 and 34) following informed consent. The workup included safety, immunological and clinical parameters. Results: Vaccinations were well tolerated. They induced a high-titer aSyn-specific antibody (Ab) response. Titer increase was associated with a reduction in aSyn plasma levels, suggesting target engagement. The Ab titer and the reduction in aSyn plasma levels were both long-lived. The boost elicited a recall-type Ab titer increase and triggered avidity maturation (factor 7.8). Abs demonstrated a high degree of selectivity for aggregated aSyn (factor 30). Moreover, they were found to preferentially react with tissue from PD brain lysates. The Movement Disorder Society-Sponsored Unified Parkinson’s Disease Rating Scale (MDS-UPDRS) score for the patient remained essentially stable throughout the observation period of 53 weeks. At the time of the boost, the symptomatic PD therapy was simplified to Levodopa/Carbidopa/Entacapone 100/25/200 mg four times a day, amounting to an LEDD of 532 mg. This put an end to the symptoms of the impulse control disorder. Conclusions: Results obtained suggest that this new class of vaccines may yield Ab responses comparable in magnitude and target avidity to the therapeutic setting of monoclonal Abs. TRB-001 is currently being translated to a randomized, placebo-controlled Phase 1B study.

## 1. Introduction

Parkinson’s disease (PD) is a progressive neurodegenerative disorder characterized by motor and non-motor symptoms. Pathological hallmarks include degeneration of nigrostriatal dopaminergic neurons and neuronal inclusions of misfolded, fibrillar alpha-synuclein (aSyn), so-called Lewy bodies (intracytoplasmic) and Lewy neurites (axonal) in specific central and peripheral nervous system regions. Current therapies are limited to symptom management, and no disease-modifying treatments are approved [1]. Consequently, there is a critical need for new therapeutic strategies. While TRB-001 targets neurodegenerative rather than infectious disease, it is still directly relevant to this special issue. Alpha-synuclein functions as an innate immune mediator upregulated by infection stimuli and interferon signaling, placing it at the intersection of host defense and neuronal biology [2]. The finding here that active immunotherapy can engage and reduce peripheral aSyn in vivo has implications beyond PD, informing how immune activation, whether pathogen- or vaccine-driven, may shape neurological outcomes.

Immunotherapies targeting aSyn represent a leading strategy for developing disease-modifying agents for PD [3]. This field includes passive immunotherapy, which involves administering pre-formed monoclonal antibodies (e.g., Prasinezumab: humanized Immunoglobulin G1 Ab targeting linear epitope amino acids (aa) 118–125 of the C-terminal region of aSyn); and active immunization, which stimulates the body’s immune system to generate a long-lasting antibody response (e.g., PD01A/ACI-7104.056: mimotope approach, target: C-terminus of aSyn, aa 110–130, UB312: target: C-terminal aSyn, aa 118–130 [4,5]). Whilst passive immunization has advantages in terms of control and specificity, it also involves significant drawbacks such as high costs and the requirement for frequent administration. The advantages of active immunization include long-lasting immune memory, fewer administrations, lower production costs, and broader access. Immunotherapy approaches to PD have faced significant hurdles, leading to the discontinuation of some programs, although in late 2025, Roche announced the advancement of Prasinezumab to Phase 3 following two successful Phase 2 studies [6]. While one of the studies missed its primary endpoint (*p* = 0.065), the results provided clear and convincing information on biological endpoints, study population and duration, and clinical efficacy measures across disease domains. In December 2025, AC Immune presented promising interim results from its Phase 2 trial. The results suggest that ACI-7104, an optimized formulation of its predecessor PD01A, stabilizes both disease-relevant biomarkers and clinical measures, indicating a clinical meaningful benefit for early-stage PD patients [7].

WISIT (Win the skin immune system trick) is an investigational therapeutic vaccine platform. It is currently being developed for PD to address the critical need for effective immunotherapy [8]. WISIT vaccines are conjugates of three elements: B-cell peptides, T-helper moieties, and beta-glucan. The B-cell peptides represent the target structure of the Ab response. Cross-Reacting Material 197 (CRM_197_) is the prototype carrier protein used as a source of T-cell help. Beta-glucan targets the dectin-1 receptor on antigen-presenting cells, resulting in increased vaccine uptake and activation of the APCs. WISIT vaccines are delivered to the dermal compartment, which is rich in dendritic cells, to induce a high-titer, high-avidity Ab response against aggregated aSyn. In animal models, WISIT vaccines surpass conventional vaccines by an order of magnitude in terms of both antibody titer and avidity [8]. Abs induced by TRB-001 are highly selective for pathological, i.e., aggregated forms of aSyn, and they are thought to prevent the propagation of aSyn pathology [8].

A phase Ib clinical study of the WISIT lead candidate TRB-001 is currently in preparation. However, special patient interest led to the treatment of a single patient with early-stage PD under a named patient use (NPU) program (early disease onset, impulse control disorder under conventional PD therapy). We report here on safety and tolerability, as well as biological-, immunological- and clinical response, in this first-in-human case involving the WISIT candidate vaccine TRB-001.

## 2. Case Presentation

The Patient: A 33-year-old Caucasian man (height: 170 cm, body weight: 83 kg) was diagnosed with PD in January 2017, at the age of 26, when he presented with impaired fine motor skills in his right hand. In 2024, according to Hoehn & Yahr, his disease stage was 1, and his MDS-UPDRS total score was 7. At the time, his symptomatic therapy was complex and required a Levodopa equivalent daily dose (LEDD) of 940 mg. Furthermore, it was complicated by impulse control disorder (cravings, gambling, punding, hours of playing computer games and shopping for food) and oedema of the lower extremities because of treatment with dopamine agonists. He has provided written informed consent for both treatment with TRB-001 and the publication of this case report.

Vaccine description and administration: TRB-001 consists of a short peptide representing the C-terminus of aSyn (aa 110–130), CRM197, a genetically detoxified form of diphtheria toxin protein, as a T-cell activating element and a beta-glucan. It was manufactured by Biosynth (Berlin, Germany). Administrations were carried out via intradermal injection using a 26-gauge needle in the upper extremities, alternating between arms. The patient received three priming immunizations of TRB-001 (dose: 100 µg total protein) intradermally on 17 September, 15 October, and 12 November 2024. A subsequent boost immunization was administered on 13 May 2025 (week 34). The patient was monitored for safety and for immunological, biological and clinical parameters.

Safety and Tolerability: TRB-001 was well tolerated. No systemic adverse events were reported. Local reactions were limited to mild, transient swelling (1 day) at the injection site following the third immunization only.

**Figure 1:**
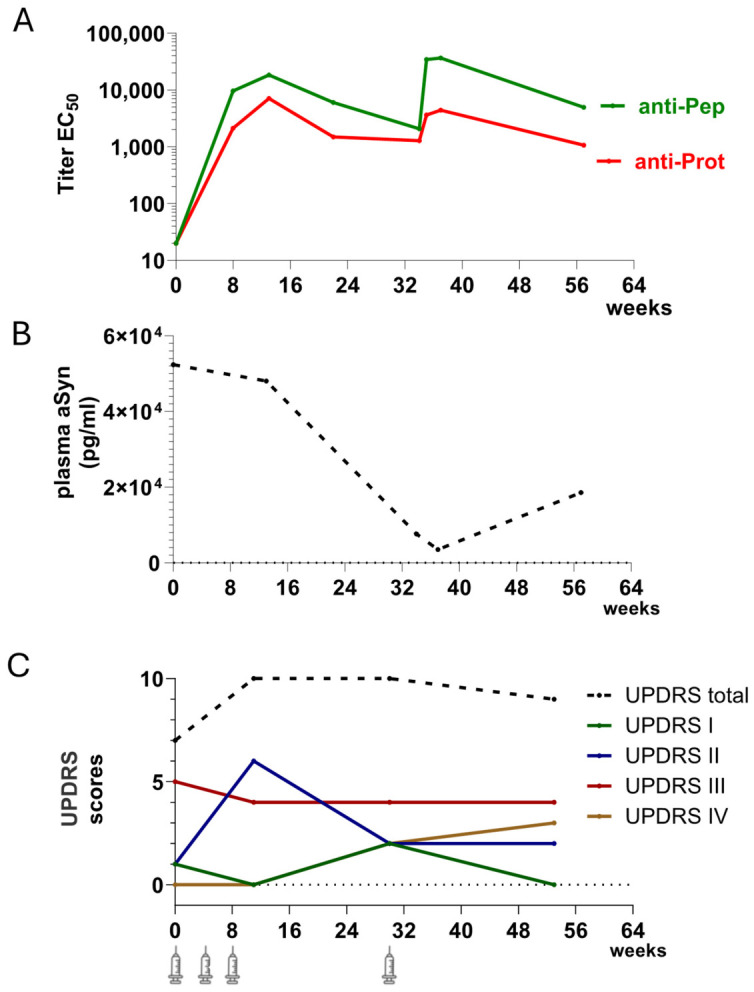
Immunological, biological and clinical outcomes. (**A**) Titers of Abs specific for the peptide component (green) of the vaccine and aSyn protein (red). A standard ELISA, validated to specifically detect IgG Abs, was used to measure Ab titers. Serum samples, collected at the indicated time points, were serially diluted (1:10 dilution steps). ELISA plates were coated with a synthetic aSyn peptide or recombinant human aSyn protein. Subsequently, diluted serum samples were added to the plates in duplicates. Peptide- and aSyn protein-specific Abs were detected with biotinylated anti-human IgG and a subsequent color reaction using Streptavidin–Peroxidase and Tetramethylbenzidin. Titers were calculated as EC50 values using Prism^®^ 10.4 following non-linear regression analysis (four-parameter logistic fit function). (**B**) Serum levels of aSyn. aSyn levels were measured using the LEGEND MAX^TM^ Human α-Synuclein ELISA kit (Biolegend, San Diego, CA, USA) according to the manufacturer’s instructions. (**C**) Clinical course: total and partial MDS-UPDRS scores. MDS-UPDRS was administered at baseline and at weeks 10, 34 and 53. The data are presented as the total score (dashed line) and the scores for parts I–IV. Syringes reflect the immunization timepoints.

**Figure 2:**
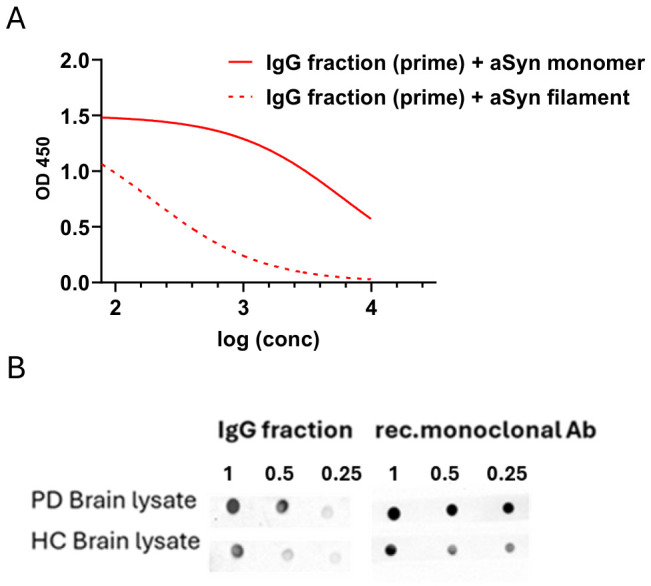
Exploratory outcomes. (**A**) Selectivity of TRB-001-induced antibodies for aggregated aSyn. To measure the selective binding and the binding strength of serum anti-aSyn antibodies to aggregated aSyn, a serum sample collected 4 weeks after the third immunization was immunoprecipitated using a Pierce™ Protein A IgG Purification Kit (Thermo Fisher, Waltham, MA, USA) to isolate the total IgG fractions. The selective binding was analyzed by inhibition ELISA. Briefly, titrated amounts of monomeric aSyn and aSyn fibrils (both from Abcam, Cambridge, UK) were pre-incubated with the IgG-fractions. These mixtures were then added to ELISA microtiter plates densely coated with aSyn monomers. Abs bound to the immobilized antigen were analyzed, and IC50 values were calculated by Prism^®^ 10.4 following non-linear regression analysis (four-parameter logistic fit function). (**B**) TRB-001-induced antibodies preferentially react with PD brain lysates. Binding of serum anti-aSyn antibodies to human PD brain lysates was analyzed by dot blot. Decreasing amounts (1.0, 0.5 and 0.25 µg) of PD brain lysates (Novus Biologicals, Centennial, CO, USA; Human Brain Whole Tissue Lysate (Adult Whole Parkinson’s), Cat No: NB820-59407) or human brain lysates (Novus Biologicals, Human Brain Whole Tissue Lysate (Adult Whole Normal) were spotted in duplicate on nitrocellulose membranes. Binding of IgG fraction of serum collected at week 12 (i.e., 4 weeks after the 3rd immunization) or a recombinant antibody (Invitrogen™ Prasinezumab Recombinant Monoclonal Antibody; Waltham, MA, USA) to each preparation was detected using an HRP-conjugated anti-Human IgG secondary antibody and the Western Lightning ECL Pro (PerkinElmer, Shelton, CT, USA; Catalog: PK-NEL122). The blots were visualized using a UVP imager (Ultra-Violet Products Ltd., ChemiDoc-It^®^ 810 Imager; Upland, CA, USA).

Immunological and Biological Outcomes: The patient developed a strong Ab response to vaccination with TRB-001. The EC_50_ aSyn protein titer increased with each of the three priming vaccinations (Figure 1A). Titers then decreased gradually by about one log until application of the boost at week 34. The boost triggered a recall-type increase in Ab titers. The kinetics of Abs specific to the peptide component of the vaccine were similar (Figure 1A). Peripheral aSyn levels decreased by >80% from the priming phase to the boost time point (Figure 1B), providing evidence of target engagement. The boost was associated with a further reduction in total peripheral aSyn levels. Thereafter, total aSyn levels increased as aSyn-specific Ab levels decreased. The induced Abs showed high selectivity for aggregated aSyn (30-fold) over monomeric forms (Figure 2A). A pronounced avidity maturation was observed. The avidity of serum Abs for aggregated aSyn increased from 0.7 M after the priming phase to 5.5 M after the boost, representing a 7.8-fold increase in binding strength (Table 1). A dot blot analysis confirmed that the patient’s Abs recognized pathological aSyn in human PD brain lysates (Figure 2B).

Clinical Course: The following results were obtained through clinical evaluation during the 53-week follow-up period following the first TRB-001 administration: The Hoehn & Yahr stage was 1 at baseline and at week 53. The patient did not report motor fluctuations or non-motor symptoms. The treatment regimen remained stable until week 33, when it was modified due to an intractable impulse control disorder, characterized primarily by excessive gambling. The initial treatment regimen of Levodopa/Benserazide (Madopar^®^; Roche, Basel, Switzerland; 100/25 mg, 6× per day), Rotigotine (Neupro^®^, 8 mg; UCB Pharma, Brussels, Belgium) and Rasagilin (1 mg; Ratiopharm, Ulm, Germany) was replaced with Levodopa/Carbidopa/Entacapone (100/25/200 mg, 4× per day; Orion Pharma, Espoo, Finland). This modification decreased the LEDD from 940 mg to 532 mg. While these significant changes complicated interpretation of clinical outcomes, including the apparent stability of MDS-UPDRS scores, they were associated with an obvious improvement in symptoms of impaired impulse control. In fact, the patient’s weight decreased by 3.6% (from 83 kg to 80 kg), and gambling ceased to be an issue. Of note, patient well-being was the primary consideration throughout. MDS-UPDRS total score, 7 at baseline, increased to 10 at weeks 10 and 34 (boost). It was 9 at the 53-week time point (Figure 1C; dashed line).

## 3. Discussion

This case study represents the first-in-human application of the WISIT PD candidate vaccine TRB-001. Results must be contextualized, given that this is a single-patient, open-label case with no control, involving a patient with mild disease and a relatively short follow-up time. However, within these limitations, this case study indicates that TRB-001 is well tolerated and induces a strong, selective, high-titer/high-avidity Ab response that demonstrates initial evidence of target engagement.

In terms of safety, the reaction to TRB-001 administration was mild. There were no systemic reactions; neither injection/allergic reactions nor reactions such as fever, malaise or headache which are often seen with conventional adjuvants [9]. Local reactions were limited to transient swelling at the site of the third immunization. There were also no indications of loss of aSyn function despite the reduction in serum aSyn levels.

The kinetics of the peptide- and aSyn-specific Ab responses increased with each vaccination: the peptide-specific Ab response was 0.5 log stronger than the aSyn-specific response. In this patient, aSyn Ab concentrations ranged between 1.13 µg/mL after priming to 0.73 µg/mL prior to the boost. These concentrations are within the range observed after Prasinezumab infusion, even though they are at the lower end [10]. Once Ab levels were established, they remained relatively stable throughout the 53-week observation period. The antiparallel kinetics of the Ab response and the plasma aSyn suggest that the TRB-001-induced Abs mediate the substantial reduction in total plasma aSyn. It is important to note that total aSyn levels do not correlate with disease severity. However, their sustained (20 weeks) reduction is unprecedented. No other published vaccine studies have reported a lowering of peripheral aSyn levels [4,5,7].

Dot blot analyses confirmed that TRB-001 induced Abs preferentially reacted with brain lysates from PD patients compared with those from healthy controls. A similar binding pattern was obtained for the Prasinezumab mimic.

This work has several limitations. First, the results are from a single patient only. The data were generated outside of a formal clinical trial framework. This meant that there was a lack of external control measures such as monitoring. The applied assays were qualified, but they were not good laboratory practice (GLP)-grade. All evaluations were performed by investigators with a financial interest in the outcome. These included the clinical assessment of the patient.

Taken together, the available information on the patient’s clinical course, including the results of the MDS-UPDRS and Hoehn & Yahr staging, as well as the LEDD over time, is consistent with the idea that the patient’s disease is stable following immunization. However, these results are difficult to interpret because the symptomatic therapy had to be modified during the observation period due to impaired impulse control. For this reason, based on the promising case report, a clinical development program will begin in the early summer of 2026.

## 4. Conclusions

This case report documents the first-in-human use of the novel aSyn candidate vaccine TRB-001 in a patient with PD. The vaccine demonstrated a favorable safety profile and induced a strong, long-lasting Ab response characterized by high selectivity and avidity against pathological, aggregated aSyn. Data indicating that the Abs induced by TRB-001 react with pathological aSyn include (i) preferential recognition of PD brain lysates and (ii) sustained reduction in total aSyn serum levels. These are prerequisites for interfering with aSyn pathology. These analyses provide important information about target engagement, help de-risk the clinical development of TRB-001, and inform the design for the upcoming Phase 1B clinical trial.

## Figures and Tables

**Table 1:** Boost is associated with strong avidity maturation of Abs that specifically react with aSyn-aggregates. The binding strength (avidity) was analyzed with an adapted ELISA in which sodiumthiocyanate (NaSCN) was used as a chaotropic agent able to disrupt the lower affinity antigen–antibody binding. Briefly, ELISA plates were coated with aSyn filaments (Abcam, Cambridge, UK), and constant amounts of IgG fractions were added to the plates in duplicate. Following plate washing, 100 μL of a chaotropic agent was added at increasing concentrations (from 0 to 3 M) for 20 min at RT. Plates were washed again, and residual antibody binding was determined.

Avidity	NaSCN (M)
Prime	0.7 ± 0.28
Boost	5.5 ± 0.71

## Data Availability

Data available on request due to restrictions (e.g., privacy, legal).
